# Supplementary material for: FastqPuri: high-performance preprocessing of RNA-seq data
Source: BMC Bioinformatics. 2019 May 3;20:226. doi: 10.1186/s12859-019-2799-0 (PMC6500068; doi:10.1186/s12859-019-2799-0)
Supplement: Supplementary file 2 — Archive of FastqPuri. Archive containing all files needed to install and run FastqPuri v1.0.6. Date stamp March 22, 2019. (GZ 47,819 kb) [file 12859_2019_2799_MOESM2_ESM.gz › FastqPuri-1.0.6/html/struct__iparam__Sreport.html]

FastqPuri: \_iparam\_Sreport Struct Reference


FastqPuri

Public Attributes |
List of all members

\_iparam\_Sreport Struct Reference

contains Sreport input parameters
More...

`#include <init_Sreport.h>`

|  |  |
| --- | --- |
| Public Attributes | |
| char \* | inputfolder |
|  | |
| char | outputfile [MAX\_FILENAME] |
|  | |
| char \* | Rmd\_file |
|  | |

## Detailed Description

contains Sreport input parameters

## Member Data Documentation

## ◆ inputfolder

char\* \_iparam\_Sreport::inputfolder

input folder

## ◆ outputfile

char \_iparam\_Sreport::outputfile[MAX\_FILENAME]

html outputfile path

## ◆ Rmd\_file

char\* \_iparam\_Sreport::Rmd\_file

Rmd file path

---

The documentation for this struct was generated from the following file:

- include/init\_Sreport.h


---

Generated on Mon Mar 19 2018 23:42:01 for FastqPuri by  

 1.8.14
